# Assessing Dental Anxiety in Naïve Patients: Development and Validation of the Naïve Dental Anxiety Scale (NDAS)

**DOI:** 10.7759/cureus.106482

**Published:** 2026-04-05

**Authors:** Iswariya A, Swati Markandey, Samapika Routray, Ambika Sundas

**Affiliations:** 1 Dentistry, All India Institute of Medical Sciences, Bhubaneswar, IND; 2 Psychiatry, Powys Teaching Health Board, NHS Wales, Wales, GBR

**Keywords:** anxiety assessment, dental anxiety, psychometric scale development, psychometric validation, treatment-naive patients

## Abstract

Background

The existing Modified Dental Anxiety Scale (MDAS) is widely used to assess dental anxiety; however, it includes experience-based terminology that may not be appropriate for naïve (first-time) dental patients. This study aimed to validate a Naïve Dental Anxiety Scale (NDAS), adapted from the MDAS, and establish its comprehensive rationality.

Methods

A cross-sectional study was conducted among 200 dental patients visiting the outpatient department of a tertiary care hospital for the first time (termed naïve patients). The NDAS was developed as a five-item questionnaire based on anticipatory dental anxiety constructs. Content validity was assessed using an expert panel, followed by face validation and pilot testing for clarity and comprehensibility. Participants completed both the NDAS and the Modified Dental Anxiety Scale (MDAS), along with demographic details. Psychometric evaluation included internal consistency using Cronbach's alpha, corrected item-total correlations, and criterion validity assessed through Spearman's correlation with MDAS. A two-tailed p-value <0.05 was considered statistically significant.

Results

The NDAS demonstrated acceptable content validity (item-level CVI (I-CVI) ≥0.78 and scale-level CVI (S-CVI) ≥0.80). Internal consistency was good (Cronbach's α=0.82), with corrected item-total correlations ranging from 0.49 to 0.70, indicating meaningful contributions of all items. A statistically significant moderate positive correlation was observed between NDAS and MDAS scores (Spearman's ρ=0.49, p<0.001), supporting criterion validity.

Conclusion

The NDAS demonstrates good internal consistency and acceptable validity for assessing dental anxiety at the group level in naïve patients.

## Introduction

Dental anxiety, an omnipresent issue with diverse manifestations, can lead to avoidance of dental care, resulting in poor oral health and compromised treatment outcomes [1]. Factors contributing to dental anxiety include previous negative experiences, fear of pain, and fear of losing control during treatment [2,3]. Managing anxious patients is also challenging for clinicians, as reduced cooperation may require additional time and resources, potentially leading to a stressful experience for both the patient and the dentist [4].

Patients visiting the dentist for the first time (hereafter referred to as "naïve patients") may experience heightened levels of anxiety due to fear of the unknown, prior negative perceptions, or misinformation. Public exposure to dental misinformation can amplify anticipatory anxiety among first-time patients, increasing the need for instruments that capture information-related concerns [5]. While the Modified Dental Anxiety Scale (MDAS) [6] is a well-established tool, it assumes prior exposure to dental procedures, making certain terms (such as drilling, scaling, and filling) ambiguous for naïve dental patients. Consequently, anticipatory anxiety, uncertainty, and information-related concerns in this population may not be adequately captured by existing instruments.

To address this gap, a tool tailored specifically to naïve patients is required. The present study aimed to develop a Naïve Dental Anxiety Scale (NDAS) for first-time dental patients and to evaluate its psychometric properties. The validation focused on key parameters, including content validity, internal consistency, and criterion validity (as measured by concurrent validity).

## Materials and methods

A cross-sectional, questionnaire-based study was conducted in the outpatient department (OPD) of the Department of Dentistry at a tertiary care hospital in Bhubaneswar, Odisha, to assess the anxiety levels of naïve dental patients. 

Ethical approval was obtained from the Institutional Ethics Committee before the commencement of the study (T/IM-NF/Dent/25-26/80).

Inclusion and exclusion criteria

Patients visiting the dentist for the first time and patients willing to participate in the study were included in the study. The exclusion criteria was patients with prior dental treatment experience, patients with psychiatric illness or cognitive impairment, and patients currently on anti-anxiety medication.

Sample size

There is no absolute consensus on the optimal sample size for psychometric validation [7]. For questionnaire development, an item-to-respondent ratio of 5:1 to 30:1 is commonly recommended [8,9]. Applying the conservative upper bound of 30 participants per item to our five-item NDAS yields a minimum of 150 participants. However, we included a total of 200 participants with consent.

A convenience sampling method was used to recruit participants until the required sample size was achieved. Patients reporting to the OPD were asked about their previous exposure to a dentist, if any. The patients with no previous dental exposure were included in the study. Written informed consent, approved by the Institutional Ethical Committee (IEC), was obtained from all participants after they agreed to participate. 

Development of NDAS

The NDAS was initially developed as a five-item questionnaire based on constructs of anticipatory dental anxiety relevant to naïve patients. Content validity was assessed by a panel of eight experts: two psychiatrists, three psychologists, and three oral surgeons with a minimum of 10 years of academic experience; using the Content Validity Index (CVI), which demonstrated acceptable item-level and scale-level validity. Based on expert feedback, minor wording modifications were made to improve clarity and relevance.

The revised questionnaire underwent forward and backward translation to ensure linguistic accuracy, followed by face validation among 10 dental-naïve patients to assess comprehensibility and interpretability. Minor refinements were incorporated based on participant feedback.

Subsequently, internal consistency reliability was evaluated in a pilot sample of 30 patients using Cronbach's alpha and corrected item-total correlations, confirming adequate homogeneity among the items. The finalized NDAS was then administered to the full study sample for further validation.

Data collection tools

Data were collected through personal interviews using a structured questionnaire. Patients' demographics (age, gender, education, and occupation) and socioeconomic status were recorded. The Modified Kuppuswamy Scale [10] was used to assess socioeconomic status (SES) based on education, occupation, and family income. The newly developed NDAS, along with the MDAS as control, was used to assess the anxiety levels of naïve patients.

Data collection procedure

Eligible patients in the waiting area were interviewed face-to-face by trained personnel. Responses were entered directly into a Google Form using a mobile device to ensure data accuracy and minimize missing entries. 

Statistical analysis

The collected data were entered into a Microsoft Excel 2024 spreadsheet (Microsoft, Redmond, Washington) and subsequently analyzed using SPSS Statistics for Windows, Version 27.0 (IBM Inc., Armonk, NY). Data distribution was assessed using the Shapiro-Wilk test, complemented by visual inspection of histograms, normal Q-Q plots, and box plots, which indicated that the NDAS scores were not normally distributed.

Content validity was established prior to data collection through an expert panel review (n=8), to calculate the item-level CVI (I-CVI) and scale-level CVI (S-CVI), with acceptable benchmarks of ≥0.78 and ≥0.80, respectively. 

Reliability of the NDAS was assessed using internal consistency measures, including Cronbach's alpha and corrected item-total correlations. Criterion validity was evaluated using Spearman's correlation with MDAS. Additionally, agreement between NDAS and MDAS scores was explored using the intraclass correlation coefficient (ICC) and Bland-Altman analysis as supplementary measures.

NDAS scores were further compared across demographic groups using a non-parametric approach. The Kruskal-Wallis test was applied for multi-group comparisons as the distributional assumptions for parametric testing were not met. A p-value of ≤0.05 was considered statistically significant for all analyses.

## Results

A total of 200 participants were included in the study. The mean age was 30.80 ± 10.93 years, with a range of 18 to 62 years. Most participants belonged to the 18-30-year age group (115, 57.5%), followed by the 31-40-year age group (41, 20.5%), the 41-50-year age group (32, 16.0%), and those over 50 years (12, 6.0%). Regarding sex distribution, males comprised 116 (58.0%) of the participants, while females accounted for 84 (42.0%) of the participants. Educational status, postgraduates and undergraduates, each comprised 50 (25.0%) participants. This was followed by higher secondary level in 48 (24.0%), high school education in 20 (10.0%), middle school in 18 (9.0%), and primary school education in 13 (6.5%) participants, while only one participant (0.5%) was illiterate.

Based on the modified Kuppuswamy socioeconomic classification, the lower middle class formed the largest proportion with 71 (35.5%) participants, followed by the upper lower class with 60 (30.0%), the lower class with 36 (18.0%), and the upper middle class with 32 (16.0%) participants. Only one participant (0.5%) belonged to the upper socioeconomic class (Table-1).

**Table 1:** Demographic characteristics of the study subjects Table 1:Demographic characteristics of the study subjects

Variable	Category	Frequency (%)
Age group	18-30 years	115 (57.5%)
	31-40 years	41 (20.5%)
	41-50 years	32 (16.0%)
	>50 years	12 (6.0%)
Sex	Male	116 (58.0%)
	Female	84 (42.0%)
Education level	Illiterate	1 (0.5%)
	Primary school	13 (6.5%)
	Middle school	18 (9.0%)
	High school	20 (10.0%)
	Higher secondary	48 (24.0%)
	Undergraduate	50 (25.0%)
	Postgraduate	50 (25.0%)
Socioeconomic class (modified Kuppuswamy)	Upper	1 (0.5%)
	Upper-middle	32 (16.0%)
	Upper-lower	60 (30.0%)
	Lower-middle	71 (35.5%)
	Lower	36 (18.0%)

The descriptive statistics of individual items and total scores of the MDAS and the newly developed NDAS are presented in Table 2. For MDAS items, mean scores ranged from 2.28 ± 1.36 to 3.13 ± 1.53. The highest mean score was recorded for the item "If you were about to have a local anaesthetic injection in your gum, above an upper back tooth, how would you feel?" (mean 3.13 ± 1.53), whereas the lowest mean was observed for "If you were sitting in the waiting room (waiting for treatment), how would you feel?" (mean 2.28 ± 1.36). The total MDAS score demonstrated a mean of 13.34 ± 5.69, with a median of 13 (IQR: 9-18). For NDAS items, mean scores ranged from 2.42 ± 1.40 to 3.19 ± 1.62. The highest mean value was obtained for the item "I wish there were facilities to relax before an appointment" (mean 3.19 ± 1.62), while the lowest mean was noted for "I think about feeling pain while waiting for my appointment" (mean 2.42 ± 1.40). The total NDAS score showed a mean of 13.20 ± 5.48, with a median of 13 (IQR: 9-17).

**Table 2:** Descriptive statistics of item-wise and total scores MDAS - Modified Dental Anxiety Scale, NDAS - Naïve Dental Anxiety Scale

Item / score	Mean ± SD	Median (IQR)
MDAS Items		
If you went to your dentist for treatment tomorrow	2.31 ± 1.47	2 (1–3)
If you were sitting in the waiting room	2.28 ± 1.36	2 (1–3)
If you were about to have a tooth drilled	2.94 ± 1.52	3 (1–4)
If you were about to have your teeth scaled and polished	2.68 ± 1.38	3 (1–4)
If you were about to have a local anesthetic injection	3.13 ± 1.53	3 (1–5)
Total MDAS score	13.34 ± 5.69	13 (9–18)
NDAS Items		
I feel nervous while waiting for my turn in the dental set-up	2.44 ± 1.34	2 (1–3)
I am worried as I have heard negative experiences about dental visits	2.44 ± 1.38	2 (1–4)
I feel nervous because I do not know what to expect	2.71 ± 1.43	3 (1–4)
I think about feeling pain while waiting for my appointment	2.42 ± 1.40	2 (1–3)
I wish there are facilities to relax before appointment	3.19 ± 1.62	4 (1–5)
Total NDAS score	13.20 ± 5.48	13 (9–17)

The NDAS demonstrated good internal consistency, with a Cronbach's alpha of 0.82, indicating adequate homogeneity among the items (Table 3). Corrected item-total correlations ranged from 0.49 to 0.70, showing that all items contributed meaningfully to the overall scale. None of the items, when deleted, resulted in an improvement in Cronbach's alpha, confirming item stability within the scale. 

**Table 3:** Internal consistency of the Naïve Dental Anxiety Scale (NDAS)

Parameter	Value	Interpretation
Cronbach's alpha (overall)	0.82	Good internal consistency

The concurrent validity of the NDAS was examined against the MDAS, which was considered the reference instrument. A statistically significant moderate positive monotonic association was observed between NDAS and MDAS total scores (Spearman's ρ=0.49, p<0.001), indicating that participants with higher NDAS scores also tended to have higher MDAS scores. Agreement between the two scales was further evaluated using intraclass correlation analysis. A single-measure ICC (absolute agreement) of 0.47 (95% CI: 0.36-0.57; p<0.001) and an average-measure ICC of 0.64 (95% CI: 0.53-0.73; p<0.001) were obtained, indicating moderate agreement between NDAS and MDAS scores (Table 4).

**Table 4:** Concurrent validity between NDAS and MDAS ICC - Intraclass correlation coefficient; CI - Confidence intervals; MDAS - Modified Dental Anxiety Scale, NDAS - Naïve Dental Anxiety Scale

Measure	Statistic	Value
Correlation (NDAS vs MDAS)	Spearman's ρ	0.49
	p-value	<0.001 (significant)
Intraclass correlation (absolute agreement, two-way mixed model)	Single-measure ICC	0.47 (95% CI: 0.36-0.57)
	Average-measure ICC	0.64 (95% CI: 0.53-0.73)
	p-value	<0.001 (significant)
Bland-Altman analysis	Mean bias	0.135
	SD of bias	5.75
	95% limits of agreement	-11.1 to 11.4

Comparison of NDAS total scores across demographic subgroups is given in Table 5. The median NDAS score did not differ significantly across age categories (p=0.21). Participants aged 18-30 years and 31-40 years showed similar anxiety levels, while those aged 41-50 years demonstrated slightly lower median scores, and those above 50 years showed marginally higher scores; however, none of these differences reached statistical significance. No significant difference was observed between males and females (p=0.65), with both sexes exhibiting comparable mean and median anxiety scores.

**Table 5:** Comparison of NDAS total scores across demographic subgroups H - statistic analyzed by Kruskal-Wallis test; U - statistic analysed by Mann-Whitney test; NDAS - Naïve Dental Anxiety Scale

Demographic characteristics/ categories		n	Mean ± SD	Median (IQR)	Test statistic	p-value
Age group						
18-30		115	13.45 ± 5.64	14 (10-17)	H=4.578	0.21 (not significant)
31-40		41	13.39 ± 4.73	13 (10-18)		
41-50		32	11.50 ± 5.84	9.5 (5-17)		
>50		12	14.67 ± 4.91	16 (14.25-17)		
Sex						
Female		84	13.42 ± 5.96	14 (9-17)	U=4691.5	0.65 (not significant)
Male		116	13.04 ± 5.12	13 (9-17)		
Education level						
Illiterate		1	17	17	H=8.706	0.19 (not significant)
Primary school		13	12.85 ± 5.05	15 (9-17)		
Middle school		18	12.78 ± 6.23	14.5 (6-17.5)		
High school		20	15.75 ± 4.84	14.5 (13.75-20)		
Higher secondary		48	12.65 ± 5.72	12.5 (7-17)		
Undergraduate		50	14.08 ± 5.41	14 (10.25-17.5)		
Postgraduate		50	12.00 ± 5.18	13 (7.25-16)		
Socioeconomic class						
Upper		1	15 ± NA	15 (15-15)	H=9.2	0.06 (not significant)
Upper-middle		32	11.19 ± 5.74	11.5 (5-15.5)		
Upper-lower		60	13.92 ± 5.52	14 (10-18)		
Lower-middle		71	12.56 ± 5.11	13 (9-15.5)		
Lower		36	15.00 ± 5.39	16 (12-18.25)		

Across different educational levels, NDAS scores were also comparable (p=0.19). Although individuals with a high school education and illiterates appeared to show numerically higher mean scores, this pattern did not translate into statistically significant variation. Similarly, socioeconomic class was not associated with significant differences in NDAS scores (p=0.06). Participants from lower socioeconomic strata tended to demonstrate slightly higher anxiety scores compared with upper or middle categories, but this trend again remained statistically nonsignificant.

## Discussion

Our study aimed to evaluate the psychometric properties of the newly established NDAS tool, designed to identify the anxiety level of naïve patients. Our studies proposed a hypothesis that they may experience higher levels of anxiety due to fear of the unknown, past undesirable perceptions, or misrepresentation. While the MDAS is a well-established tool, it assumes prior exposure to dental procedures, making certain terms (such as drilling, scaling, and filling) ambiguous to naïve dental patients. Additionally, its concurrent validity with the MDAS was also examined, as various scales are available to assess dental anxiety [11]. Previous reports are suggestive of modifications being made to various anxiety scales to assess anxiety levels specific to the extraction procedure [12] and the surgical procedure [13]. The validity and reliability of a measuring tool, such as a scale or questionnaire, reduce "measurement errors", and the measurements that come from the measuring process will be accurate [14]. The content validity of the existing scale was established by engaging multiple specialists in dental surgery (general dentists), psychiatry (psychiatrists), and psychology (psychologists) who examined and assessed the scale's questions, grounded in theoretical principles [15]. After validating the content of the tool, the psychometric properties of NDAS were evaluated using 200 participants (116 males and 84 females) who were naïve patients. The reliability of the NDAS was primarily supported by internal consistency measures (Cronbach's alpha) [16]. Additionally, agreement with MDAS was explored using ICC, although the two instruments are not intended to be interchangeable. Our study demonstrated a significant p-value, confirming the validity of the scale.

The NDAS tool demonstrated good internal consistency, with a Cronbach's alpha value of 0.82, indicating adequate homogeneity among the items. This finding is comparable with previously validated dental anxiety instruments. Corah et al., developed one of the earliest tools for assessing dental anxiety (Corah's Dental Anxiety Scale), which has reported Cronbach's alpha values ranging from 0.80 to 0.86 across different populations [17,18]Similarly, the Modified Dental Anxiety Scale [6] (MDAS) developed by Humphris et al., has consistently demonstrated high internal consistency, with alpha values ranging from 0.85 to 0.93 in various validation studies conducted across different cultural and linguistic settings [6,19]. Comparable values have been reported in earlier validation studies of dental anxiety scales, particularly during initial scale development or when applied to specific populations [19-21].

The concurrent validity of the NDAS was supported by a statistically significant moderate positive correlation with the MDAS. This level of association is consistent with previous studies conducted by Newton et al. and Tunc et al., comparing newly developed dental anxiety measures with established instruments, where moderate correlations have been considered acceptable due to differences in scale structure, item focus, and population characteristics [20,22]. The findings indicate that while both NDAS and MDAS assess the underlying construct of dental anxiety, the NDAS may capture unique anxiety dimensions relevant to dental-naïve individuals, making our study and the tool novel. The observed moderate correlation with the MDAS suggests that while both instruments assess dental anxiety, they capture related but not identical constructs. This is expected, as the NDAS is specifically designed to evaluate anticipatory anxiety in dental-naïve individuals, whereas the MDAS primarily reflects procedure-related anxiety. Therefore, the NDAS should not be considered interchangeable with the MDAS but rather complementary in assessing distinct dimensions of dental anxiety.

It was observed that the NDAS tool scores were not significantly influenced by age, gender, educational attainment, or socioeconomic status. The absence of statistically significant differences across age groups suggests that dental anxiety, as measured by the NDAS, remains relatively stable throughout adulthood. Although participants aged 41-50 years demonstrated slightly lower anxiety scores and those above 50 years showed marginally higher scores, these variations did not reach statistical significance, indicating that age alone may not be a strong determinant of dental anxiety in this population. This finding aligns with previous literature suggesting that dental anxiety is shaped more by individual psychological factors and prior experiences than by chronological age [23]. There were no significant gender differences observed in our responses, with males and females exhibiting comparable anxiety scores. While earlier literature frequently reported higher dental anxiety among females [6], more recent studies suggest that gender differences may be diminishing, potentially due to changing sociocultural roles and increased dental awareness across sexes [24]. The current results confirm that the NDAS tool captures anxiety constructs that are not inherently gender-dependent. Educational level was also not significantly associated with NDAS scores. Although participants with lower educational attainment and those without formal education showed numerically higher anxiety levels, these differences were not statistically meaningful. This finding is correlated with studies suggesting that dental anxiety is not solely determined by educational status or health literacy, but rather by personal beliefs, fear conditioning, and cognitive appraisal of dental procedures [25,26]. The NDAS's focus on anticipatory anxiety and uncertainty may therefore transcend educational differences. Similarly, another factor, such as socioeconomic status, did not show a statistically significant association with dental anxiety, although there was a non-significant trend toward higher scores among individuals from lower socioeconomic strata. Preceding studies have suggested that financial constraints, limited access to dental care, and prior negative treatment experiences may contribute to increased anxiety in disadvantaged populations [27]. The lack of statistical significance in the present study may reflect sample characteristics or limited power to detect subtle socioeconomic effects.

These findings suggest that the NDAS functions reliably across key demographic subgroups, supporting its demographic neutrality and potential applicability in diverse adult populations. The lack of significant demographic effects reinforces the notion that dental anxiety is a multifactorial construct influenced more by individual cognitive and emotional processes than by sociodemographic characteristics alone.

Overall, the findings suggest that the NDAS demonstrates good internal consistency and acceptable validity for assessing dental anxiety, particularly in individuals with limited or no prior dental experience. However, the findings of the present study should be interpreted as preliminary, representing an initial step in the validation of the NDAS.

The study has several strengths, including its specific focus on dental-naïve patients, a clearly defined conceptual framework for item development, and the use of multiple validation approaches such as content validation and internal consistency assessment.

Despite these strengths, the study has certain limitations. The cross-sectional design precluded assessment of test-retest reliability and responsiveness to change over time. Additionally, while a moderate association with MDAS was observed, this indicates that the NDAS captures related but not identical dimensions of dental anxiety, and therefore should not be considered interchangeable with existing instruments. The study was conducted at a single center using a convenience sampling approach, which may limit generalizability. Furthermore, criterion validity was assessed only against a self-reported measure, without inclusion of behavioral or physiological indicators of anxiety.

Future studies should aim to evaluate the factor structure, test-retest reliability, and predictive validity of the NDAS across diverse clinical and cultural settings, and to examine its utility in predicting treatment-related outcomes such as dental avoidance and patient compliance.

## Conclusions

In conclusion, the NDAS demonstrates good internal consistency and acceptable validity for assessing dental anxiety at the group level in individuals with no prior dental experience. The findings support its potential utility as a research tool for evaluating anticipatory dental anxiety in naïve populations. However, further studies are required to establish its temporal stability, predictive validity, and clinical applicability before it can be recommended for individual-level screening or decision-making.

## Appendices

Questionnaires: 

1) Naive Dental Anxiety Scale (NDAS) for naïve patients

1. I feel worried while waiting for my turn in the dental setup.

2. I am worried, as I have heard negative experiences about dental visits.

3. I feel nervous because I do not know what to expect

4. I think about feeling pain while waiting for my appointment.

5. I wish there are facilities to relax before appointment. 

(Rating Scale: 1 = Strongly Disagree, 2 = Disagree, 3 = Neutral, 4 = Agree, 5 = Strongly Agree)

2) Modified Dental Anxiety Scale (MDAS) pre- and post-treatment [5]

1. If you went to your dentist for a check-up

2. If you were about to have a tooth drilled

3. If you were about to have your teeth scaled and polished

4. If you were waiting in the dental chair

5. If you were about to have an injection in your gum

Scoring (1-5): 1=not anxious;2=slightly anxious; 3= fairly anxious; 4= very anxious; 5= extremely anxious
